# Protective effect of Tat fused HPCA protein on neuronal cell death caused by ischemic injury

**DOI:** 10.1016/j.heliyon.2023.e23488

**Published:** 2023-12-16

**Authors:** Hyun Jung Kwon, Hyo Young Jung, Soo Young Choi, In Koo Hwang, Dae Won Kim, Min Jea Shin

**Affiliations:** a Department of Biomedical Science and Research Institute of Bioscience and Biotechnology, Hallym University, Chuncheon 24252, Republic of Korea; b Department of Veterinary Medicine, Institute of Veterinary Science, Chungnam National University, Daejeon 34134, Republic of Korea; c Department of Anatomy and Cell Biology, College of Veterinary Medicine, Research Institute for Veterinary Science, Seoul National University, Seoul 08826, Republic of Korea; d Department of Biochemistry and Molecular Biology, Research Institute of Oral Sciences, College of Dentistry, Gangneung-Wonju National University, Gangneung 25457, Republic of Korea

**Keywords:** Tat-HPCA, Brain ischemia, Oxidative stress, Protein transduction domain, Protein therapy

## Abstract

**Background:**

Bain ischemia is a disease that occurs for various reasons, induces reactive oxygen species (ROS), and causes fatal damage to the nervous system. Protective effect of HPCA on ischemic injury has not been extensively studied despite its significance in regulating calcium homeostasis and promoting neuronal survival in CA1 region of the brain.

**Objective:**

We investigate the role of HPCA in ischemic injury using a cell-permeable Tat peptide fused HPCA protein (Tat-HPCA).

**Methods:**

Western blot analysis determined the penetration of Tat-HPCA into HT-22 cells and apoptotic signaling pathways. 5-CFDA, AM, DCF-DA, and TUNEL staining confirmed intracellular ROS production and DNA damage. The intracellular Ca^2+^ was measured in primary cultured neurons treated with H_2_O_2_. Protective effects were examined using immunohistochemistry and cognitive function tests by passive avoidance test and 8-arm radial maze test.

**Results:**

Tat-HPCA effectively penetrated into HT-22 cells and inhibited H_2_O_2_-induced apoptosis, oxidative stress, and DNA fragmentation. It also effectively inhibited phosphorylation of JNK and regulated the activation of Caspase, Bax, Bcl-2, and PARP, leading to inhibition of apoptosis. Moreover, Ca^2+^ concentration decreased in cells treated with Tat-HPCA in primary cultured neurons. In an animal model of ischemia, Tat-HPCA effectively penetrated the hippocampus, inhibited cell death, and regulated activities of astrocytes and microglia. Additionally, Cognitive function tests show that Tat-HPCA improves neurobehavioral outcomes after cerebral ischemic injury.

**Conclusion:**

These results suggest that Tat-HPCA might have potential as a therapeutic agent for treating oxidative stress-related diseases induced by ischemic injury, including ischemia.

## Graphical abstract

**Figure:**
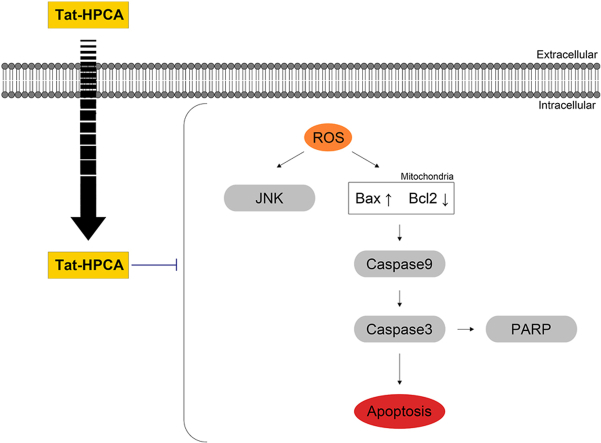

## 1 Introduction

Reactive oxygen species (ROS) generated by oxidative stress play a secondary electron role in various cellular processes including cell proliferation and differentiation [[1], [2], [3]]. In the nervous system, ROS generated by oxidative stress are known to be major risk factors for neurological disorders [[4], [5], [6], [7]], particularly in cerebral ischemia where neurons in the Cornu Ammonis-1 (CA1), which is the first region in the hippocampal circuit area of the hippocampus are highly vulnerable to oxidative stress, leading to severe neuronal cell death [[8], [9], [10]].

Hippocalcin (HPCA), a member of nerve calcium sensor (NCS) proteins, is abundantly expressed in pyramidal neurons of the CA1 region of the hippocampus. HPCA possesses a calcium/myristoryl switch that maintains tonicity of calcium, which can regulate various neural functions, including gene expression within cells, neuronal growth, differentiation, survival, and apoptosis. Furthermore, function of HPCA in regulating calcium plays an important role in inhibiting neuronal differentiation and apoptosis [[11], [12], [13]]. Aberrant activity of HPCA can increase vulnerability to oxidative stress-induced damage and result in neuronal death and neurodegeneration. Despite its involvement in oxidative stress-induced brain diseases including stroke, research on HPCA remains limited. As HPCA operates intracellularly, delivering HPCA into cells poses a significant challenge. Consequently, we used protein infiltration techniques to make the HPCA fusion protein. The novel HPCA fusion protein surmounts the inherent in research methodologies, facilitating its potential applications, including research and therapeutic development.

Delivering large molecules such as proteins into tissues and cells is a challenging task due to various reasons. Protein infiltration techniques can be utilized to overcome this challenge. Cell-penetrating peptides (CPPs) are short peptides composed of 5–30 amino acids. They can pass through cell membranes, tissues, and even the blood-brain barrier (BBB) without needing specific receptors [14,15]. Many studies have reported that CPP-conjugated therapeutic proteins can be introduced into cells and tissues with protective efficacy against various diseases both *in vitro* and *in vivo* [[16], [17], [18]].

The objective of the present study was to investigate protective effect of cell-penetrating Tat-HPCA protein against oxidative stress-induced hippocampal neuronal cell death in HT-22 mouse hippocampal neuronal cell line and animal models of cerebral ischemia/reperfusion injury.

## 2 Materials and methods

### 2.1 Expression and purification of Tat-HPCA protein

Human HPCA was amplified by PCR with the sense primer 5′-CTCGAGATGGGCAAGCAG-3′ which contained an *Xho*I restriction site and the antisense primer 5′-GGATCCTCAGAACTGGGA-3′ which contained a *Bam*HI restriction site. The resulting PCR products were ligated into a TA cloning vector (pGEM®-T easy vector; Promega Corporation, Madison, WI, USA) according to the manufacture's protocol and subcloned in the Tat expression vector which has been described in a previous study [19]. The constructed control-HPCA and Tat-HPCA plasmids were transformed into *Escherichia coli (E. coli)* BL21 cells and proteins expression were induced by 0.5 mM isopropyl-β-d-thiogalactopyranoside (IPTG; Duchefa, Haarlem, Netherlands) at 30 °C for 16 h. Control-HPCA and Tat-HPCA protein was purified using a Ni^2+^-nitrilotriacetic acid Sepharose affinity column (Qiagen, Valencia, CA, USA) and PD-10 column chromatography (Amersham, Braunschweig, Germany).

### 2.2 HT-22 cell culture and transduction of Tat-HPCA protein

HT-22, mouse hippocampal cells were grown in Dulbecco's minimum essential medium (DMEM) containing 10 % fetal bovine serum (FBS) (Lonza BioWhittaker, MD, USA) and antibiotics (100 μg/ml streptomycin, 100 U/ml penicillin) at 37 °C under humidified conditions of 95 % air and 5 % CO_2_.

The cells were treated with various concentrations of Tat-HPCA protein (0.5–3 μM) for 1 h or with 3 μM for various time periods (15–60 min). To determine the intracellular stability of Tat-HPCA protein, the cells were treated with Tat-HPCA for 1 h and were incubated further for 1–60 h. The cells were harvested to perform Western blot analysis.

### 2.3 Western blot analysis

Protein extraction was performed using cell lysis buffer (RIPA; ELPIS BIOTECH, Daejeon, Korea) according to the manufacturer's instructions. Equal amounts of proteins in cell lysates were separated by 15 % SDS-PAGE and transferred to a nitrocellulose membrane (PALL, AZ, USA). The membrane was blocked with TBS-T (25 mM Tris-HCl, 140 mM NaCl, 0.1 % Tween 20, pH 7.5) buffer containing 5 % non-fat dry milk for 1 h. The blocked membrane was incubated with primary antibodies overnight at 4 °C, followed by incubation with horseradish peroxidase-conjugated secondary antibodies (1:10,000; Cell signaling technology, Danvers, USA) at room temperature for 1 h. Primary antibodies were diluted as 1:1000 and purchased CST. The proteins were detected by chemiluminescence according to the manufacturer's instructions (Millipore, MA, USA).

### 2.4 Cell viability assay

HT-22 cells were treated with various concentrations of Tat-HPCA protein (0.5–3 μM) for 1 h and then incubated with hydrogen peroxide (H_2_O_2_, 0.1 mM) for 2 h. WST-1 cell viability assay kit (Daeil Lab service Co., Seoul, Korea) was used according to the manufacturer's protocol. Cell viability was defined as the % of untreated control cells.

### 2.5 5-CFDA, AM

5-Carboxyfluorescein Diacetate, Acetoxymethyl Ester (5-CFDA, AM; Invitrogen, Carlsbad, CA) which is hydrolyzed by intracellular esterases, was used as a probe of viable cells and according to the manufacturer's protocol. HT-22 cells were treated with 3 μM Tat-HPCA and 0.1 mM H_2_O_2_ for 1 h. Cells were exposed to 1 μM 5-CFDA, AM for 20 min at 37 °C. Images were observed under a fluorescence microscopy (Eclipse 80i, Nikon, Tokyo, Japan) and the fluorescence intensity was measured using a Fluoroskan ELISA plate reader (Labsystems Oy, Helsinki, Finland) excitation at 485 nm and emission at 538 nm.

### 2.6 Analysis of ROS and DNA fragmentation levels

Intracellular ROS levels and DNA fragmentation were detected using 2′,7′-Dichlorofluorescein diacetate (DCF-DA; Sigma-Aldrich, St. Louis, MO, USA) and Terminal deoxynucleotidyl transferase-mediated dUTP nick-end labeling (TUNEL; Roche Applied Science, Penzberg, Germany) according to the manufacturer's protocol, respectively. HT-22 cells were incubated with Tat-HPCA protein (3 μM) for 1 h and then treated with 0.1 mM H_2_O_2_. The cells were washed twice with PBS and incubated with 10 μM DCF-DA for 20 min and TUNEL for 60 min. Each fluorescent images were obtained by fluorescence microscopy.

### 2.7 Primary culture of neuron

The hippocampus was dissected from E16 mice embryo, and briefly triturated. Cell was seeded on poly-l-ornithine (Sigma-Aldrich, St Louis, Mo, USA)-precoated 100 mm culture dish at a seeding density of 2 × 10^5^ cells. The cells were grown in Neurobasal medium with 2 % B-27 Plus Supplement (Sigma-Aldrich, St Louis, Mo, USA) at 37 °C in a humidified atmosphere of 5 % CO_2_ in air.

### 2.8 Intracellular Ca^2+^ measurement

The intracellular Ca^2+^ was measured using calcium detection kit according to the manufacturer's instructions (Abcam, Cambridge, MA, USA). Briefly, cells grown on 60 mm dishes were homogenized and centrifuged at 21,000 g for 15 min at 4 °C. The supernatant was collected and reacted with chromogenic reagent. The absorbance of formed chromophore was measured at 575 nm.

### 2.9 Experimental animals and treatment

Male gerbils (65–75 g) were obtained from the Experimental Animal Center at Hallym University. The animals were housed at a constant temperature (23 °C) and relative humidity (60 %) with a fixed 12 h light/dark cycle and free access to food and water. All experimental procedures involving animals and their care conformed to the Guide for the Care and Use of Laboratory Animals of the National Veterinary Research and Quarantine Service of Korea and were approved by the Hallym Medical Center Institutional Animal Care and Use Committee (Hallym 2021-55).

The ischemia animal model was carried out as previously described [[20], [21], [22]]. Briefly, the animals were anesthetized, common carotid arteries were isolated, freed of nerve fibers, and occluded with non-traumatic aneurysm clips. After 5 min occlusion, the aneurysm clips were removed. The reperfusion was observed using an ophthalmoscope. Tat-HPCA protein were intraperitoneally injected 30 min after reperfusion (each group, *n* = 7). The Mortality rate during the 7 days after surgery was 20 % in sham group, 24 % in vehicle group, 22.5 % in Tat-HPCA treated group, and 23.3 % in HPCA treated group.

### 2.10 Immunohistochemical analysis

Immunohistochemistry was performed as described in previous studies. Briefly, Brain tissue samples were obtained at 7 days after ischemia-reperfusion. The sections were incubated with diluted cresyl violet acetate (CV; Sigma-Aldrich, Merck KGaA), rabbit anti-glial fibrillary acidic protein (GFAP; 1:1000; Chemicon International) and rabbit anti-ionized calcium-binding adapter molecule 1 (Iba-1, 1:500; Wako, Osaka, Japan) for 48 h at 4 °C. Images were captured and analyzed using an Olympus DP72 digital camera and DP2-BSW microscope digital camera software.

### 2.11 Passive avoidance test (PAT)

PAT was conducted considering previous studies to evaluate short-term memory following ischemic injury [22]. In short, we used the Gemini Avoidance System (GEM 392) (San Diego Instruments, San Diego, CA, USA), which consists of dark and light sections communicating by a vertical sliding door between the two sections. The training was performed, and the substantive trial was conducted 20 min after the training. In the training, the gerbil was placed in the light section and allowed to freely explore both sections for 1 min while the vertical door was opened. When the gerbil entered the dark section, the door was closed, and an electric foot shock (0.5 mA for 5 s) was given to the gerbil from the steel grid on the floor. For the substantive trial, the gerbil was placed in the light section, and the latency time to enter the dark section was recorded within 3 min.

### 2.12 8-Arm radial maze test (8-ARMT)

Radial eight-arm maze test measures spatial learning and working memory [23]. The number of correct and incorrect choices was analyzed using the radial 8-arm maze test. A small container filled with 20 μl of water was placed at the end of each arm in the radial 8-arm maze device. The rats were deprived of water for 48 h and then allowed to explore and drink water for 8 min. After 24 h, the test section recorded the correct number of times it reached each arm and sought water for over 8 min. Re-entering the previously visited arm was counted as an error and the number of times all water in each arm was found was recorded as an error.

### 2.13 Statistical analysis

Data are expressed as the mean ± SEM of three experiments. Differences between groups were analyzed by ANOVA followed by a Bonferroni's post-hoc test. Statistical significance was considered at *P* < 0.05 and *P* < 0.01.

## 3 Results

### 3.1 Purification and transduction of Tat-HPCA into HT-22 cells

**Fig. 1:**
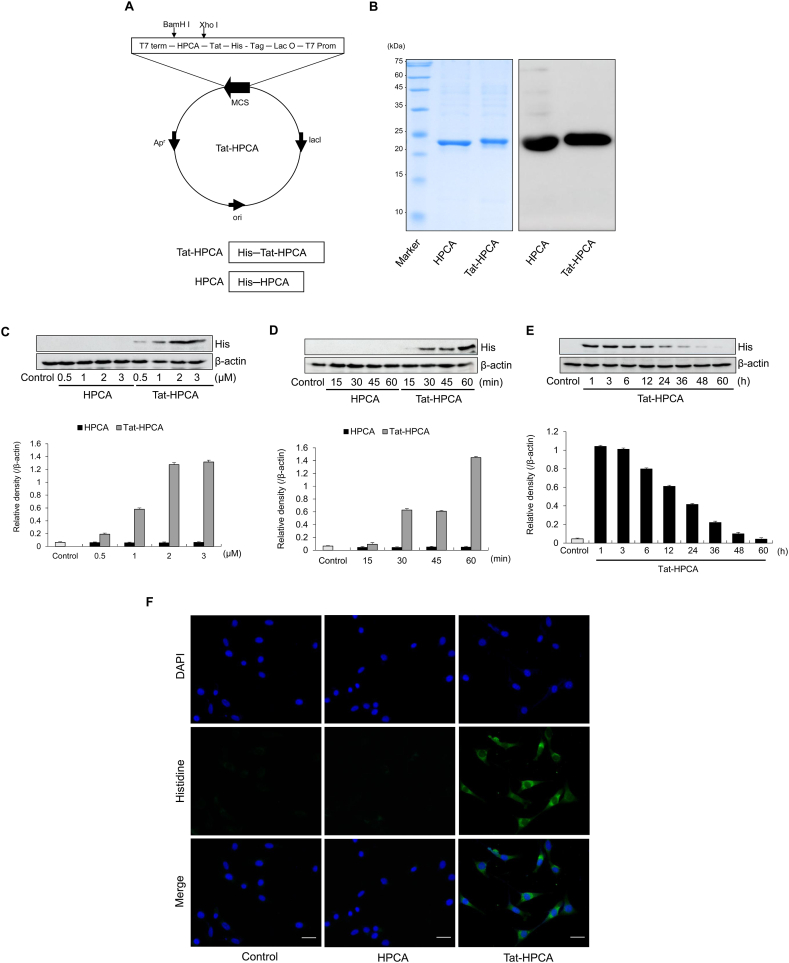
Purification and Transduction of Tat-HPCA protein into HT-22 cells. The constructed map of Tat-HPCA based on the pET15b vector that contains 6 histidine residues (A). Expressed and purified Tat-HPCA proteins were separated by 15 % SDS–PAGE and the protein was confirmed by Western blot analysis with an anti-rabbit polyhistidine antibody (B). Cells were treated with Tat-HPCA proteins (0.5–3 μM) for 1 h (C), or cells were treated with Tat-HPCA proteins (3 μM) for 15–60 min (D). Intracellular stability of transduced Tat-HPCA protein. After Tat-HPCA proteins (3 μM) transduced into the HT-22 cells, the cells were incubated for 1–60 h (E). Transduced Tat-HPCA protein levels were analyzed by Western blotting and the intensity of the bands was measured by densitometer. The distribution of transduced Tat-HPCA protein was examined by confocal fluorescence microscopy (F). Scale bar = 20 μm.

Human HPCA gene was cloned into a Tat expression vector containing His-tags to generate a cell-permeable Tat-HPCA protein (Fig. 1A). As a control, the HPCA gene was also fused with His-tags without a Tat peptide. Both Tat-HPCA and control-HPCA proteins were overexpressed in *Escheirchia coli* BL21 (DE3) cells using IPTG induction and purified through Ni-NTA affinity chromatography. Purified proteins were then verified through SDS-PAGE and Western blotting utilizing an anti-His antibody (Fig. 1B). To assess the efficacy of Tat-HPCA transduction into HT-22 cells, cells were treated with Tat-HPCA protein at various concentrations (0.5–3 μM) for 1 h or with Tat-HPCA protein at a constant concentration (3 μM) for different time periods (15–60 min). As depicted in Fig. 1C and D, levels of transduced Tat-HPCA increased in a dose- and time-dependent manner, whereas control-HPCA failed to enter cells. Additionally, intracellular stability of the transduced Tat-HPCA protein was sustained for 36 h in HT-22 cells (Fig. 1E). Distribution of the transduced Tat-HPCA protein was further confirmed using DAPI and Alexa Fluor 488, revealing that transduced Tat-HPCA proteins were predominantly located in the cytoplasm (Fig. 1F). Conversely, no control-HPCA protein was detected. These findings demonstrate successful transduction of Tat-HPCA protein into HT-22 cells.

### 3.2 Effects of Tat-HPCA protein on H_2_O_2_-induced ROS damage in cells

**Fig. 2:**
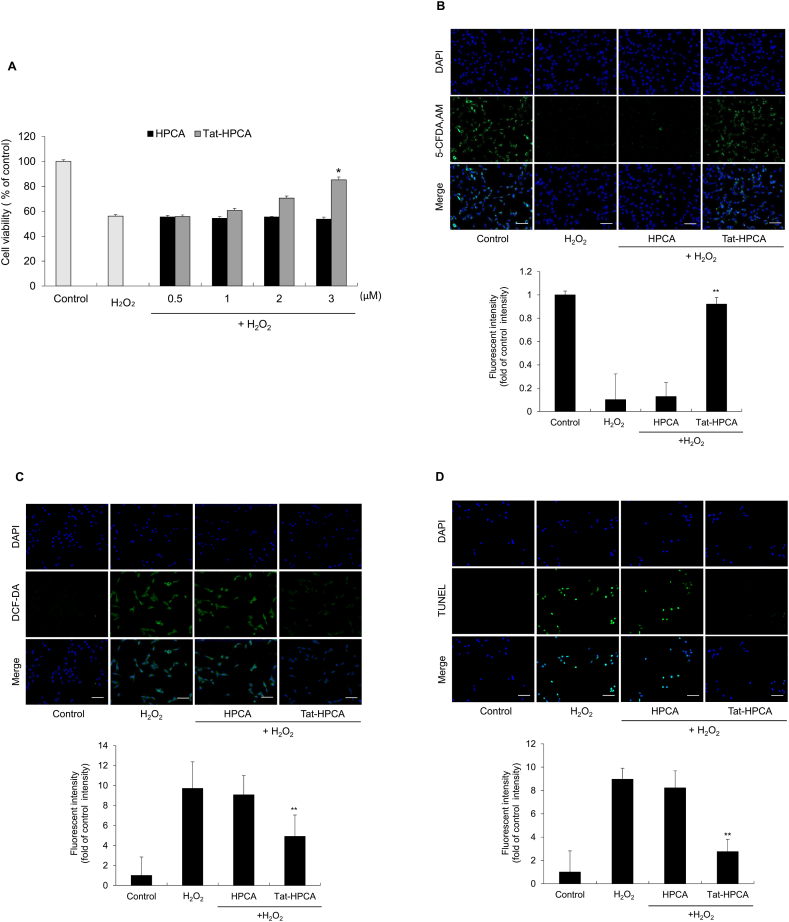
Effect of Tat-HPCA protein on H_2_O_2_-induced cellular toxicity. HT-22 cells were pretreated with Tat-HPCA (0.5–3 μM) for 1 h and exposed to H_2_O_2_ (100 μM) 2 h. Cell viabilities were assessed by WST-1 assay (A) and 5-CFDA, AM (B). Intracellular ROS levels were measured using DCF-DA staining (C). DNA fragmentation was detected by TUNEL staining (D). The fluorescence intensity was measured by an ELISA plate reader. *∗P < 0.01*, compared with H_2_O_2_-treated cells.

Effects of Tat-HPCA on H_2_O_2_-induced cell death in HT-22 cells were evaluated. Cells were treated with Tat-HPCA or control-HPCA at various concentrations (0.5–3 μM), followed by exposure to 100 μM H_2_O_2_. Cell viability assessed using WST-1 assay was found to increase (up to 85 %) by Tat-HPCA in a dose-dependent manner. However, control-HPCA had no significant effect on cell survival (Fig. 2A). Fluorescence measurements using 5-CFDA, AM also showed a decrease in cell viability of H_2_O_2_ treated cells and control-HPCA treated cells in comparison with those of untreated and Tat-HPCA treated cells (Fig. 2B). To investigate effects of Tat-HPCA on oxidative stress, levels of ROS generation and DNA fragmentation were measured using DCF-DA and TUNEL assays (Fig. 2C–D). Results showed that H_2_O_2_ treatment significantly increased intracellular ROS and DNA fragmentation, while Tat-HPCA treatment significantly reduced these levels. However, there was no significant difference in intracellular ROS or DNA fragmentation between H_2_O_2_ treated and control-HPCA treated cells. These findings suggest that transduced Tat-HPCA protein can protect cells against H_2_O_2_-induced cellular damage.

### 3.3 Inhibition effects of Tat-HPCA on H_2_O_2_-induced apoptosis signal pathway and Ca^2+^ increasing

**Fig. 3:**
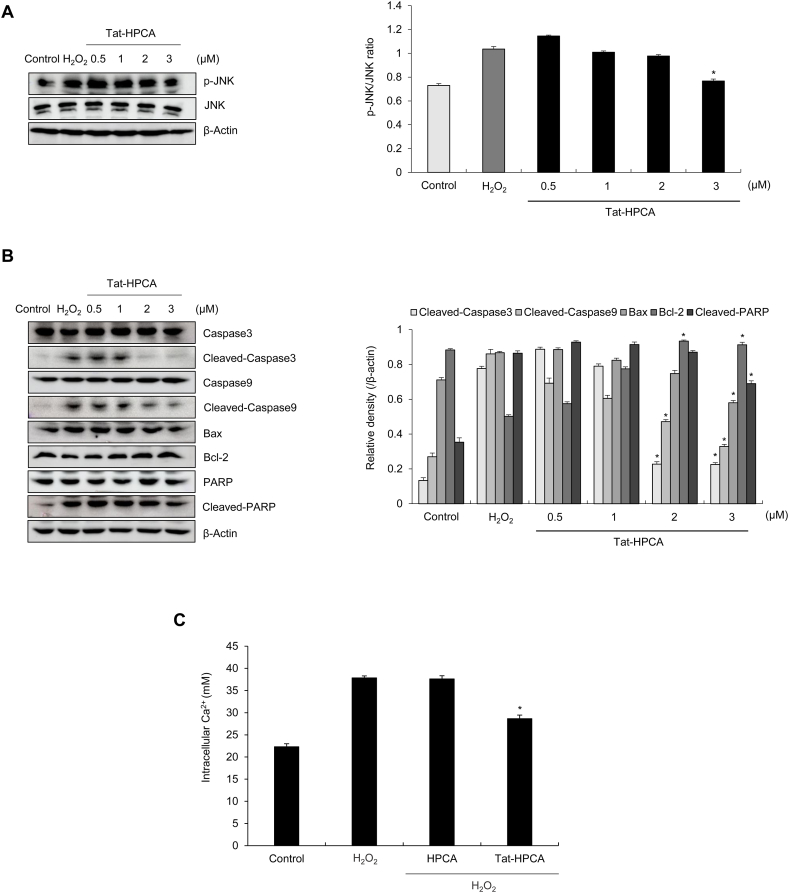
Inhibitory effects of Tat HPCA protein against H_2_O_2_-induced cellular signaling pathways in HT-22 cells. Tat-HPCA (3 μM) for 1 h before being treated with H_2_O_2_ (100 μM). H_2_O_2_-induced JNK activation was examined using Western blot analysis (A). The expression levels of caspase-3, caspase-9, PARP, Bax and Bcl-2 were determined by Western blot analysis (B). The level of intracellular Ca^2+^ was measured in cell culture medium (C). Effects of H_2_O_2_ and Tat-HPCA on calcium concentration (mM). Band intensity was measured by densitometer. The bars in the figure represent the mean ± SEM obtained from 3 independent experiments. *∗P < 0.01*, compared with H_2_O_2_-treated cells.

The impact of Tat-HPCA on H_2_O_2_-induced apoptosis of HT-22 cells was studied using Western blot analysis. JNK, a member of the MAPK pathway, plays a significant role in both extrinsic and intrinsic apoptotic pathways. Its activation is characterized by sequential phosphorylation [[23], [24], [25]]. Thus, effects of Tat-HPCA on JNK pathway were evaluated (Fig. 3A). Results showed that JNK was phosphorylated in HT-22 cells exposed to H_2_O_2_, whereas its phosphorylation was reduced by Tat-HPCA in a concentration-dependent manner. However, control-HPCA had no significant effect on JNK phosphorylation. Additionally, expression levels of cleaved-caspase-3, cleaved-caspase-9, and cleaved-PARP were upregulated in H_2_O_2_ treated cells. However, such upregulations were reversed by Tat-HPCA in a concentration-dependent manner. Moreover, Tat-HPCA decreased the expression of pro-apoptotic protein Bax, but increased the expression of anti-apoptotic protein Bcl-2 (Fig. 3B). These results indicate that Tat-HPCA has potential to mitigate H_2_O_2_-induced apoptosis.

We also verified whether the apoptosis protection effect of Tat-HPCA is associated with its calcium-regulating function. Fig. 3C shows that Tat-HPCA reduces the elevated calcium concentration attributed to ischemic damage in primarily cultured neurons. These findings suggest that HPCA, as a calcium sensor protein, is implicated in both calcium regulation and the occurrence of ischemic injury.

### 3.4 Tat-HPCA prevents neuronal cell death in an ischemic animal model

**Fig. 4:**
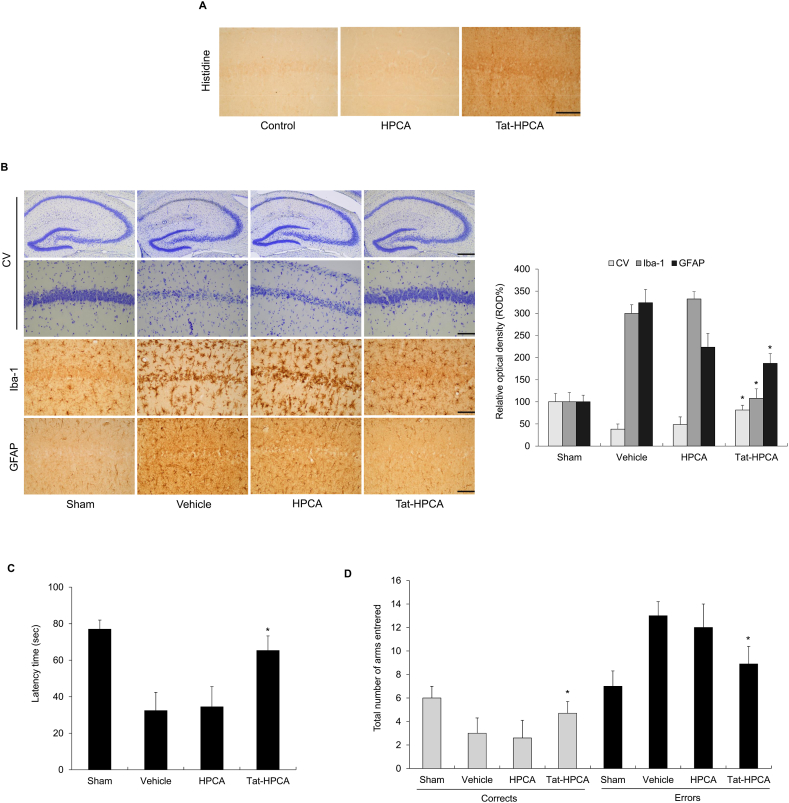
Protective effect of Tat-HPCA protein in ischemic injury animal model. Gerbils were treated with a single injection Tat-HPCA protein (2 mg/kg) before ischemia-reperfusion and killed after 7 days. Transduced Tat-HPCA protein was analyzed by immunostaining using anti-His antibody (A). The hippocampus was stained with CV, Iba-1 and GFAP in sham-, vehicle-, HPCA and Tat-HPCA-treated animals 7 days after I/R (B). Scale bar = 400 and 50 μm. Latency time by passive avoidance (PAT) and mean numbers of errors in 8-arm radial maze test (8-ARMT) in ischemic injury animal model. In the Tat-HPCA-treated group, a significantly delayed latency time in PAT is recorded compared with that in the vehicle groups (C). In the 8-ARMT, the Tat-HPCA-treated group showed a significantly improved number of correct and error choices compared to the vehicle group (D). *∗P < 0.05*, compared to the vehicle group. (each group, *n* = 7).

The potential of Tat-HPCA to cross the blood-brain barrier (BBB) and inhibit neuronal cell death in an ischemic animal model was investigated. Immunohistochemistry using a histidine antibody demonstrated efficient transduction of Tat-HPCA protein into the hippocampal CA1 region of the animal brain (Fig. 4A). To examine effects of transduced Tat-HPCA protein on neuronal cell damage and activation of microglia and astrocytes, staining was performed for CV, Iba-1, and GFAP (Fig. 4B). Results showed a marked increase in CV-positive neurons in the Tat-HPCA-treated group compared to that in the vehicle- or control-HPCA treated group. GFAP-immunoreactive astrocytes were also significantly reduced in CA1 regions of the Tat-HPCA-treated group compared to those in other groups. Moreover, Iba-1-immunoreactive microglia were evident and aggregated in vehicle- or HPCA treated group, but significantly reduced in the Tat-HPCA-treated group to levels similar to the sham group. These findings suggest that Tat-HPCA transduction into the brain can significantly prevent neuronal cell death in the CA1 region following ischemic injury through reduction of microglia and astrocyte activation.

To investigate cognitive function, the gerbils in each group received a passive avoidance test (PAT) and an 8-arm radial maze test (8-ARMT) to examine spatial memory function. The vehicle group showed cognitive impairment in the PAT and 8-ARMT compared to the sham group. As shown in Fig. 4C, the latency time for rats staying in the light chamber decreased in the vehicle group but increased in the Tat-HPCA-treated group. Moreover, the Tat-HPCA-treated group increased the number of correct choices and decreased the number of errors compared to the vehicle group (Fig. 4D). These results indicate that the blockade of Tat-HPCA attenuates ischemic injury-induced cognitive impairment.

## 4 Discussion

Brain ischemia/reperfusion (I/R) injury can lead to various neurological disorders by causing nervous system damage and brain damage. During cerebral I/R injury, oxidative stress creates excessive ROS, causing lipid peroxidation and DNA damage, which leads to neuronal cell death [26].

As a member of the neuron calcium sensor family, HPCA can interact with proteins such as NAIP (Neuronal apoptosis inhibitory protein) and MLK2 (mixed lineage kinase 2) to perform various functions in neurons [13,27]. HPCA can perform a range of functions, including cell death, neuronal dendrite proliferation, gene transcription, long-term depression, and regulation of cyclic nucleotide signaling. Recent studies have shown that HPCA protein has inhibitory functions against ROS generated by oxidative stress [28]. Additionally, several studies have shown that HPCA has protective effects against neurodegenerative diseases such as Huntington's and Parkinson's [27,29], although its role in brain ischemia has not been investigated yet.

CPP (cell-penetrating peptides) are well-known for overcoming limitations of drug delivery. Numerous studies have demonstrated CPP-conjugated proteins could by efficiently up taken by cells, tissues, and even the blood-brain barrier (BBB), thereby exhibiting efficacy [[30], [31], [32]]. In this study, we investigated effects of a cell-penetrating Tat-HPCA protein on oxidative stress-induced neuronal cell death and animal model.

Tat-HPCA was found to penetrate HT-22 cells in a time-effective and concentration-dependent manner. It was stably maintained within cells for 36 h. The effect of Tat-HPCA on cell survival was measured using WST-1 assay and 5-CFDA, AM staining after inducing cell death with hydrogen peroxide (H_2_O_2_). Results showed that exposure to Tat-HPCA significantly increased cell survival rate from 55 % to 85 %, as confirmed by protective green fluorescence seen in 5-CFDA, AM stained Tat-HPCA treatment group. Furthermore, the role of Tat-HPCA in reducing oxidative stress and DNA fragmentation was evaluated by inducing oxidative stress with H_2_O_2_ and measuring ROS production and DNA fragmentation through DCF-DA and TUNEL staining, respectively. Tat-HPCA treatment significantly reduced ROS production and DNA fragmentation, consistent with observed inhibition of neuronal cell death [[33], [34], [35]]. These findings demonstrate that Tat-HPCA protein can reduce oxidative stress and prevent cell death caused by oxidative stress by inhibiting ROS levels within cells.

C-Jun *N*-terminal kinase (JNK) is a key signaling protein that regulates cellular survival, immune responses, and apoptosis. It is activated by various stressors including oxidative stress [[24], [25], [36]]. Several studies have reported that activated JNK is inhibited by HPCA, which binds to MLK2 [27]. Thus, effects of Tat-HPCA protein on JNK signaling in oxidative-induced cell death were investigated. As shown in Fig. 3A, Tat-HPCA significantly reduced phosphorylation levels of JNK. This result implies that Tat-HPCA can reduce JNK phosphorylation, exhibiting a protective effect against ROS-mediated cell death. Moreover, the role of Tat-HPCA in the regulation of cell death-related signaling proteins such as Caspase, Bax, and Bcl-2 was investigated to analyze the mechanism of cell death signaling. Results showed that Tat-HPCA effectively regulated these signaling proteins to inhibit cell death, consistent with other studies on the protective mechanism of neural cell death [[37], [38], [39]]. Moreover, we investigated calcium levels within HPCA in primary cultured neurons. As shown Fig. 3C, the Ca^2+^ concentration decreased in cells treated with Tat-HPCA. This suggests that the function of Tat-HPCA as a calcium sensor protein contributed to the protective effect against cerebral ischemia.

The present study investigated the efficacy of Tat-HPCA protein in protecting against brain ischemia/reperfusion injury in animal models. Microglial and astrocyte activations are primary causes of brain diseases including ischemia. They have an impact on neuronal cell death [[40], [41], [42]]. Effects of Tat-HPCA on microglial and astrocyte activations in ischemic injury animal models were evaluated using Iba-1 and GFAP immunohistochemical markers for microglia and astrocytes, respectively. Tat-HPCA showed protection against microglial and astrocyte activations in the CA1 area of the hippocampus, indicating that animals were protected from ischemic injury. Additionally, cognitive function tests show that Tat-HPCA improves neurobehavioral outcomes after cerebral ischemic injury. These results suggest that Tat-HPCA protein has neuroprotective effects against neuronal cell death in ischemic animal models, although further research is needed to accurately determine the underlying mechanisms involved to develop ischemic injury therapeutic agents.

## 5 Conclusions

Tat-HPCA protein can effectively penetrate cells and tissues, including HT-22 cells and the hippocampus CA1 region. It can also modulate oxidative stress-induced cell death signals via JNK phosphorylation, thus preventing cell apoptosis. Therefore, we propose that Tat-HPCA has the potential to be used as a therapeutic for various oxidative stress-mediated diseases, including those related to cerebral ischemia. This highlights the protective effect of Tat-HPCA against neuronal cell death in oxidative stress conditions.

## Funding statement

This research was supported by Basic Science Research Program {NRF–2019R1A6A1A11036849 to Soo Young Choi and NRF-2021R1F1A1048079 to Dae Won Kim} through the 10.13039/501100003725National Research Foundation of Korea (NRF) funded by the Korea government (MSIT).

## Data availability statement

Data associated with the study has not been deposited into a publicly available repository and data will be made available on request.

## CRediT authorship contribution statement

**Hyun Jung Kwon:** Methodology, Investigation, Formal analysis, Data curation, Conceptualization. **Hyo Young Jung:** Methodology, Formal analysis. **Soo Young Choi:** Methodology, Formal analysis, Data curation. **In Koo Hwang:** Methodology, Formal analysis, Data curation. **Dae Won Kim:** Writing - review & editing, Methodology, Investigation, Funding acquisition, Formal analysis, Data curation, Conceptualization. **Min Jea Shin:** Writing - review & editing, Writing - original draft, Project administration, Methodology, Investigation, Formal analysis, Data curation, Conceptualization.

## Declaration of competing interest

The authors declare that they have no known competing financial interests or personal relationships that could have appeared to influence the work reported in this paper.
